# A multi-arm multi-stage clinical trial design for binary outcomes with application to tuberculosis

**DOI:** 10.1186/1471-2288-13-139

**Published:** 2013-11-14

**Authors:** Daniel J Bratton, Patrick PJ Phillips, Mahesh KB Parmar

**Affiliations:** 1 Medical Research Council Clinical Trials Unit at University College London, 125 Kingsway, London, UK

**Keywords:** Multi-arm, Multi-stage, Tuberculosis, Binary outcome, Adaptive design, Treatment selection

## Abstract

**Background:**

Randomised controlled trials are becoming increasingly costly and time-consuming. In 2011, Royston and colleagues proposed a particular class of multi-arm multi-stage (MAMS) designs intended to speed up the evaluation of new treatments in phase II and III clinical trials. Their design, which controls the type I error rate and power for each pairwise comparison, discontinues randomisation to poorly performing arms at interim analyses if they fail to show a pre-specified level of benefit over the control arm. Arms in which randomisation is continued to the final stage of the trial are compared against the control on a definitive time-to-event outcome measure. To increase efficiency, interim comparisons can be made on an intermediate time-to-event outcome which is on the causal pathway to the definitive outcome.

**Methods:**

We adapt Royston’s MAMS design to binary outcomes observed at the end of a fixed follow-up period and analysed using an absolute difference in proportions. We apply the design to tuberculosis (TB), an area where many new drugs are in development, and demonstrate how it can greatly accelerate the evaluation of new TB regimens. We use simulations to support the extensions to the methodology and to investigate the amount of bias in the estimated treatment effects of arms in which randomisation is ceased at the first interim analysis and arms which continue to the final stage of the trial.

**Results:**

The proposed seamless phase II/III TB trial designs are shown to greatly reduce sample size requirements and trial duration compared to conducting separate phase II and III trials. The bias in the estimated treatment effects for the definitive outcome is shown to be small, especially when treatment selection is based on an intermediate outcome or when a reanalysis is performed at the planned end of the trial after all recruited patients have completed follow-up.

**Conclusions:**

The proposed designs are practical and could be used in a variety of disease areas. They hold considerable promise for speeding up the evaluation of new treatments particularly in TB where many new regimens will soon be available for testing in phase II and phase III trials.

## Background

In recent years the pace of drug development in some disease areas has rapidly increased. Despite this, there has been a slowdown in the rate of new therapies reaching patients [1]. This is largely due to the increasing cost and inefficiency of the drug development process and that most new treatments have no clear benefit over standard care. As a result, the US Food and Drug Administration (FDA) has called for a new ‘product development toolkit’ for improving the predictability and efficiency of clinical trials.

Due to the acceleration in drug development it is now more common for multiple new treatments to be simultaneously available for testing in clinical trials. In tuberculosis (TB), for example, there are currently at least ten new or repurposed drugs in the clinical pipeline [2-4]. Since TB is treated using a combination of drugs rather than monotherapy, the number of regimens that could potentially be assessed in phase II or III trials in the near future is vast. Evaluating each new treatment against a control in separate two-arm trials will not only require a huge amount of resources but may deny patients access to the most effective, simplest and shortest new regimens as early as possible. Innovative trial designs which are able to efficiently assess multiple new treatments simultaneously are therefore urgently needed.

In discussing such an issue, Phillips et al. [5] have suggested the use of the multi-arm multi-stage (MAMS) design described by Royston et al. [6]. This particular type of MAMS design, which controls the type I error rate and power for each pairwise comparison, streamlines treatment evaluation in two ways. First, comparing multiple new regimens against a single, common control arm removes the need for separate control arms in multiple two-arm trials and reduces the overall required sample size. For example, comparing four experimental arms in parallel to a single control (five-armed trial) reduces the required sample size by 37% compared to four separate two-arm trials if no adjustments for multiple testing are made. In general, comparing *K* experimental arms to a single control reduces the overall sample size by a factor of (*K*−1)/2*K* compared to *K* separate two-arm trials [7].

Secondly, the analysis of a MAMS trial is conducted in stages. At the end of each stage recruitment to an experimental arm is stopped if it fails to show sufficient evidence of an advantage over the control arm (lack-of-benefit). If an experimental arm passes the final stage of the study then it is deemed to be superior (or non-inferior, depending on the objective) to the control. The efficiency of this procedure can be greatly increased by using an outcome in the intermediate stages which is observed earlier and on the causal pathway to the final, definitive outcome of the trial, although it does not necessarily have to be a surrogate [8,9]. For example, the MAMS design may be used for a seamless phase II/III trial where the intermediate outcome is that used in a phase II trial while a phase III outcome is of primary interest in the final stage. Using an intermediate outcome in this way allows interim analyses to be conducted sooner and so recruitment to poorly performing arms can be stopped much earlier than if the primary outcome of the trial was used throughout. If a suitable intermediate outcome is unavailable then the MAMS design may still be used, for example, as a standalone phase II or III trial. The multi-stage aspect of the design removes the need to recruit a fixed sample size to all experimental arms in the trial and can further reduce the sample size compared to multi-arm fixed sample designs.

The sample size calculation for the MAMS design described by Royston et al. [6] is only applicable to time-to-event outcomes where a hazard ratio is typically the summary statistic used to compare an experimental treatment against a control. It is therefore applicable to trials in oncology, for example, where time to an event such as death is often used as a primary endpoint. The STAMPEDE trial in prostate cancer [8] for instance, uses this particular type of MAMS design. However, if it is to be more widely used in other disease areas then the methodology needs extending to other types of outcome.

In TB, a commonly used outcome measure for phase II trials is the absolute difference in the proportion of patients who have a negative culture status eight weeks after commencing therapy [10-12]. In phase III, the absolute difference in the proportion of patients who either fail to respond to their allocated treatment or relapse after completing treatment is the outcome of choice and is usually assessed 1-2 years after randomisation [13]. In this paper we use these examples as motivation for extending the design to binary intermediate and definitive outcomes observed at the end of fixed follow-up periods and analysed using an absolute difference in proportions. The benefits of this design and issues surrounding it are explored and simulation studies using examples in a TB context are used to verify the methodology and to investigate the bias in treatment effect estimates.

## Methods

### Overview of proposed design

Let *I* denote the intermediate and *D* the definitive outcome of a MAMS trial. The same null and alternative hypotheses are used for all experimental arms to allow interim analyses to be conducted simultaneously. The sample size requirement is therefore the same for each pairwise comparison in each stage and so the sample size calculation can be developed by first considering a single experimental arm, *E*, against a control, *C*.

For a MAMS trial with *s* stages, let $\pi_{i}^{E}$ and $\pi_{i}^{C}$ denote the true event rates in the *i*th stage of the trial in an experimental arm and the control arm respectively (*i*=1,…,*s*). If the same outcome is used throughout the trial (*I*=*D*) then $\pi_{i}^{E}$ and $\pi_{i}^{C}$ are constant for all *i*. If the intermediate and definitive outcomes differ (*I*≠*D*) the values $\pi_{s}^{E}$ and $\pi_{s}^{C}$ correspond to the true treatment effects for the definitive outcome and $\pi_{i}^{E}$ and $\pi_{i}^{C}$ are constant for all *i*<*s* and correspond to the intermediate outcome.

The null and alternative hypotheses for the true absolute risk difference at the *i*th interim analysis, $\theta_{i}=\pi_{i}^{E}-\pi_{i}^{C}$, are, without loss of generality,

$$\begin{array}{lll} H_{0} & :\;\theta_{i}\leq \theta_{i}^{0},\;i=1,\ldots ,s\; & \;\\ H_{1} & :\;\theta_{i}>\theta_{i}^{0},\;i=1,\ldots ,s\; & \; \end{array}$$

The value $\theta_{i}^{0}$ is constant for all *i* if the same outcome measure is used throughout the trial (*I*=*D*). Otherwise $\theta_{s}^{0}$ corresponds to the definitive outcome and $\theta_{i}^{0}$ is constant for all *i*<*s* for the intermediate outcome. In superiority analyses, $\theta_{i}^{0}$ is usually taken to be 0 to represent no difference under the null hypothesis. By contrast, non-inferiority analyses use a value of $\theta_{i}^{0}$ to represent that *E* is slightly inferior to *C* under the null hypothesis.

Having specified the null and alternative hypotheses above, the one-sided significance level, *α*_*i*_, and power, *ω*_*i*_, for each pairwise comparison is chosen for each stage of the trial. It is recommended to use a high power in each stage, for example, 90% or 95%, in order to achieve high overall power for the trial [6]. A large significance level should be used in the first stage to allow the first interim analysis to occur early on in the trial. Over subsequent stages significance levels are decreased to avoid stages becoming redundant. For trials with 6 or fewer stages Royston et al. [6] suggest a ‘rule of thumb’ of *α*_*i*_=0.5^*i*^ for stages *i*=1,…,*s*−1 and *α*_*s*_=0.025 in the final stage to mimic a conventional two-sided test at the 5% level. However, further research by Barthel et al. [14] and Choodari-Oskooei et al. [15] have suggested using a significance level between 0.2 and 0.3 in the first stage to reduce bias and error rates.

At the *i*th interim analysis recruitment continues to experimental arms whose treatment effect estimate on the intermediate outcome is significant at the 100*α*_*i*_*%* level, otherwise consideration is given for ceasing further randomisations to it. If the treatment effect estimate on the definitive outcome is significant at the 100*α*_*s*_*%* level in the final analysis then the experimental treatment is declared superior to the control arm (or non-inferior, depending on the objective).

### Sample size calculation

Since each stage has its own significance level and power we can effectively consider each stage as an independent trial. Common formulae can therefore be used to obtain the required sample size for each interim analysis. For example, the required sample size for the control arm in the *i*th interim analysis, $n_{i}^{C}$, can be calculated using [16,17]

(1) $$n_{i}^{C}=\frac{\left(z_{1-\alpha_{i}}+z_{\omega_{i}})[\;A\pi_{i}^{C}\left(1-\pi_{i}^{C}\right)+\pi_{i}^{1}\left(1-\pi_{i}^{1}\right)\right]}{A{\left(\theta_{i}^{1}-\theta_{i}^{0}\right)}^{2}}$$

where $\theta_{i}^{1}$ is the minimum effect that one would like to find with high probability for the outcome in the *i*th stage (usually the minimally clinically important difference), $\pi_{i}^{1}=\pi_{i}^{C}+\theta_{i}^{1}$ is the target event rate in the experimental arm under *H*_1_, *z*_*k*_ is the *k*th percentile of the standard normal distribution and the *E*:*C* allocation ratio is *A*:1 so that *A* patients are randomised to each experimental arm for every patient allocated to control.

For a MAMS trial with *K*_*i*_ experimental arms present in stage *i* the total sample size required for the *i*th interim analysis is then

(2) $$n_{i}=(1+K_{i}A)n_{i}^{C}.$$

### Consequences of a fixed follow-up period

Often in clinical trials, patients are followed-up for a set period of time after randomisation before outcomes are observed. For example, in phase II TB trials the endpoint of interest is often culture status 8 weeks after randomisation. An immediate consequence of delayed observations is that patients may withdraw or become lost-to-follow-up before their outcome is observed. If it is likely that outcome data will not be available for some proportion of patients, *λ*_*i*_, on the outcome in the *i*th stage of the study, then the required sample size calculated using (2) should be multiplied by 1/(1−*λ*_*i*_) to maintain the desired level of power for a complete-case analysis. It should be noted that such an analysis assumes that missing data occur completely at random which might not be plausible, in which case appropriate imputation techniques should be applied [18].

For simplicity, the loss-to-follow-up rate *λ*_*i*_ is assumed to be constant throughout the trial for each outcome. One might normally expect a higher loss rate for *D* than *I* as it requires a longer follow-up period, however, it may be easier to obtain the former, particularly if it can be ascertained from medical records (for example, death), in which case a lower attrition rate on *D* may be a plausible assumption.

Another consequence of delayed observations is that interim analyses cannot take place as soon as the required sample size has been recruited and randomised. Since recruitment is continuous, the delay in obtaining data on an outcome means that there will be patients at each interim analysis who have been recruited to the trial but who have yet to have their outcome observed. For example, if the follow-up period is six months and the recruitment rate is 100 patients per year, then an extra 50 patients will be recruited to the trial but will not complete follow-up by the time of the database freeze for the interim analysis.

These extra patients who are randomised to arms which are subsequently dropped at the interim analysis will also not contribute towards any future interim analyses. However, for reasons concerning bias (see ‘Results’ section) these patients should still be followed up for their intermediate and definitive outcomes and included in a final analysis of their allocated arm against all control arm patients randomised concurrently at the planned end of the trial.

The delay in starting the next stage of the trial caused by data cleaning, analysis, various committee meetings and changing the randomisation codes (if necessary) increases the number of patients allocated to an arm which may imminently be dropped from the trial. A possible solution to avoid randomising patients during this interval and the follow-up period is to suspend recruitment once the required sample size has accrued and then recommence it at the start of the next stage. However, this is not recommended since it is likely to prolong the duration of the trial by slowing the overall recruitment rate [5].

### Calculating the stage durations

The total expected delay, *γ*_*i*_, between recruiting the last of the *n*_*i*_ patients required for the *i*th interim analysis and the beginning of the next stage of the trial incorporates the follow-up period for the outcome plus the additional delays caused by the analysis. Denoting by *N*_*i*_ the total number of patients recruited to the arms remaining in the study by the end of stage *i*, the number of patients that need to be recruited during stage *i* for the upcoming interim analysis, ${ñ}_{i}$, is

$${ñ}_{i}=n_{i}-\frac{{\text{AK}}_{i}+1}{{\text{AK}}_{i-1}+1}N_{i-1}(1-\lambda_{i})$$

where *N*_0_=0 and *K*_*i*_ is the number of active experimental arms in the *i*th stage of the study. It follows that the duration of stage *i* is

$$d_{i}=\frac{{ñ}_{i}}{r_{i}(1-\lambda_{i})}+\gamma_{i}$$

where *r*_*i*_ is the overall recruitment rate in the *i*th stage (assumed to be constant within each stage).

The cumulative number of patients allocated to all treatment arms still recruiting at the end of each intermediate stage is then

$$N_{i}=r_{i}d_{i}+\frac{{\text{AK}}_{i}+1}{{\text{AK}}_{i-1}+1}N_{i-1}\;i=1,\ldots ,s-1.$$

In the final stage recruitment to the trial may be terminated as soon as *N*_*s*_=*n*_*s*_/(1−*λ*_*i*_) patients have been allocated to the remaining treatment arms. It would not be necessary to continue recruitment beyond this point, since there are no more analyses planned beyond the final analysis.

The stage end-times, *t*_*i*_, are obtained by summing the durations of all preceding stages; $t_{i}=\overset{i}{\underset{k=1}{\sum }}d_{k}$. These values are particularly useful as they roughly predict when interim analyses will occur and so help to organise data monitoring and trial steering committee meetings in advance.

### Probability of passing each stage

Using similar formulae to Royston et al. [6] the probabilities of an experimental arm passing the first *i* stages of a MAMS trial are

$$\begin{array}{lll} A_{i} & =\Phi_{i}(z_{\alpha_{1}},\ldots ,z_{\alpha_{i}};R_{i}^{0})\;\text{under}\;H_{0}\; & \;\\ \Omega_{i} & =\Phi_{i}(z_{\omega_{1}},\ldots ,z_{\omega_{i}};R_{i}^{1})\;\text{under}\;H_{1}\; & \; \end{array}$$

where $\Phi_{i}(;R_{i}^{h})$ is the *i*-dimensional multivariate normal distribution function with correlation matrix $R_{i}^{h}\;(h=0,1)$. The (*j*,*k*)th entry of $R_{i}^{h}$ is the correlation between the treatment effects in stages *j* and *k* under hypothesis *H*_*h*_. The calculation of these correlations is outlined in the appendix.

Clearly, *A*_1_=*α*_1_ and *Ω*_1_=*ω*_1_. The most important values are *A*_*s*_ and *Ω*_*s*_ which are the overall type I error rate, *α*, and power, *ω*, respectively for a single experimental arm compared to the control. Note that here we have only calculated the pairwise type I error rate and power — the issue of familywise error rates for trials with multiple experimental arms is raised in the discussion.

Other values of interest, particularly in a seamless phase II/III design, are *A*_*s*−1_ and *Ω*_*s*−1_ which denote the probability of continuing recruitment to an arm in the final (phase III) stage of the trial under *H*_0_ and *H*_1_ respectively. Phase III trials are often resource intensive and lengthy and the same may be true for the final stage of a MAMS trial if the intermediate and definitive outcomes differ. Therefore it is important to have a reasonably small value of *A*_*s*−1_ and a large value of *Ω*_*s*−1_ to increase the chance of only recruiting patients to effective treatments in the final stage.

As shown in the appendix, the calculation of *A*_*s*_ and *Ω*_*s*_ when *I*≠*D* requires estimates of either the probability of a patient experiencing both outcomes or the probability of experiencing the definitive outcome given they have had the intermediate outcome (positive predictive value, PPV). The latter is arguably easier to specify as it only requires an assumption for a single outcome, *D* (given that *I* has occurred), rather than two (both *I* and *D*). As the correlations between treatment effects, and therefore *A*_*s*_ and *Ω*_*s*_, increase as either of these probabilities tend to 1, we recommend slightly overestimating them to obtain a conservative estimate of the pairwise type I error rate.

### Application to tuberculosis

To illustrate how this design might be applied and assess the benefits of the MAMS design in a TB setting we used the methodology above to calculate the sample size for phase II and seamless phase II/III two-arm two-stage TB trials. Seamless designs are an effective tool for streamlining treatment evaluation as they remove the long interlude between phases which is required for presenting the phase II results and for designing, approving and funding the phase III trial. Furthermore, by reusing phase II patients in the analysis of the phase III outcome, seamless designs offer greater efficiency over the traditional approach of conducting phase II and III trials separately [19].

The phase II two-arm two-stage designs were based upon a recent study by Dorman et al. [10] that substituted moxifloxacin for isoniazid in the standard TB regimen during the intensive phase (first two months) of treatment. The outcome in this study was culture status (a marker for whether a patient has TB or not) 8 weeks after randomisation and was also used as the basis for the intermediate outcome in the seamless phase II/III designs. The phase III aspect was based on the ongoing REMox TB trial (controlled comparison of two moxifloxacin containing treatment shortening regimens in pulmonary tuberculosis) that investigates the effect of two four month regimens against the standard six month regimen on relapse rates 18 months after randomisation [20]. This trial uses a Bonferroni-adjusted one-sided significance level of 1.25% for each treatment arm to ensure the overall type I error rate is no higher than 2.5%. For this example we considered only one experimental arm from REMox and thus used a one-sided significance level of 2.5%. The designs of these standalone phase II and phase III trials are summarised in Table 1.

**Table 1:** Design parameters for phase II and III TB trials

**Design**	**Study**		Overall*
**Parameter**	**Phase II**	**Phase III**	
Primary outcome	Negative	Non-	
	culture status	failure/relapse	
Follow-up length	8 weeks**	18 months**	
Significance level (1-sided)	2.5%	2.5%	0.1%
Power	80%	85%	68%
Control arm event rate	75%	90%	
Treatment effect under *H*_0_	0%	-6% (NI margin)	
Target treatment effect (*H*_1_)	13%	0%	
Allocation ratio (*E*:*C*)	1:1	1:1	
Attrition rate	15%	20%	
Required sample size***	320	1122	1442

Design parameters for a phase II (based on Dorman et al. [10]) and a phase III (based on REMox [20]) TB trial. *Calculated assuming independence between trials. **An additional 6 week delay is typically required to determine culture status. ***Sample sizes estimated using equations (1) and (2). NI = non-inferiority.

Examples of two-arm two-stage phase II and phase II/III TB trials were generated using a conventional significance level (2.5%) and power (90%) in the final stage. Significance levels of 20% and 50% and powers of 90% and 95% were explored in the first stage. Delays of 4 and 14 weeks for observing a patient’s culture status after randomisation were used to explore their effect on the efficiency of a trial. The latter was chosen as it is the current delay in observing a patient’s culture status after randomisation due to the 8 week follow-up period plus 6 week wait for detecting absence of TB (in liquid medium). A 4 week delay was also chosen as it is not yet certain whether culture status at 8 weeks is an appropriate intermediate outcome for long-term relapse, and observing it after 4 weeks may be more suitable. Furthermore, the 6 week wait is unlikely to exist in future as techniques for immediate detection of TB are developed [21] and so observing status at 4 weeks may represent the shortest possible delay for this outcome. Examples of two-arm two-stage phase II and phase II/III TB trial designs based on these parameters are shown in Tables 2 and 3 respectively and discussed in the ‘Results’ section.

**Table 2:** Examples of two-arm two-stage phase II TB trials

**Design**	**Stage (*i*)**	*α*_*i*_	***ω***_***i***_	**Length of f/u = 4 weeks**				Length of f/u = 8 weeks*				***ρ***	***A***_***i***_	***Ω***_***i***_	**Fixed**
				***n***_***i***_	***N***_***i***_	***t***_***i***_	**ESS |** ***H***_**0**_	***n***_***i***_	***N***_***i***_	***t***_***i***_	**ESS** ***|H***_***0***_				**sample size**
(i)	1	0.5	0.90	56	96	0.48	262	56	134	0.67	281	0.39	0.500	0.900	360
	2	0.025	0.90	364	428	2.30		364	428	2.49			0.021	0.826	
(ii)	1	0.5	0.95	94	140	0.70	284	94	178	0.89	303	0.51	0.500	0.950	398
	2	0.025	0.90	364	428	2.30		364	428	2.49			0.023	0.870	
(iii)	1	0.2	0.90	156	214	1.07	257	156	252	1.26	287	0.65	0.200	0.900	381
	2	0.025	0.90	364	428	2.30		364	428	2.49			0.020	0.843	
(iv)	1	0.2	0.95	214	282	1.41	311	214	320	1.60	342	0.77	0.200	0.950	414
	2	0.025	0.90	364	428	2.30		364	428	2.49			0.023	0.883	

Characteristics of two-arm two-stage TB trials where *I* = *D* = culture status observed 4 or 14 weeks after randomisation. The fixed sample sizes correspond to fixed sample designs with pairwise alpha *A*_2_ and power *Ω*_2_. Key: for stage *i*, *α*_i_ = significance level, *ω*_i_ = nominal power, *n*_i_ = total sample size required for analysis *i*, *N*_i_ = cumulative number of patients recruited by the end of stage *i*, *t*_i_ = predicted timing (in years) of the end of stage *i* assuming a recruitment rate of 200 patients/year, ESS |*H*_0_ = expected sample size under the null hypothesis, *ρ* = correlation between stages, *A*_i_ = probability of passing stage *i* under *H*_0_, *Ω*_i_ = probability of passing stage *i* under *H*_1_. *Plus an additional 6 week delay to determine culture status.

**Table 3:** Examples of two-arm two-stage phase II/III TB trials

**Design**	**Stage(*i*)**	***α***_***i***_	***ω***_***i***_	***n***_***i***_	***N***_***i***_	***t***_***i***_	**ESS |** ***H***_**0**_	***ρ*** **|** ***H***_**0**_	***ρ*** **|** ***H***_**1**_	***A***_***i***_	***Ω***_***i***_
(v)	1	0.5	0.90	56	134	0.67	723	0.10	0.08	0.500	0.900
	2	0.025	0.90	1050	1312	3.84				0.015	0.813
(vi)	1	0.5	0.95	94	178	0.89	745	0.12	0.11	0.500	0.950
	2	0.025	0.90	1050	1312	4.00				0.015	0.857
(vii)	1	0.2	0.90	156	252	1.26	464	0.16	0.14	0.200	0.900
	2	0.025	0.90	1050	1312	4.28				0.008	0.815
(viii)	1	0.2	0.95	214	320	1.60	518	0.19	0.16	0.200	0.950
	2	0.025	0.90	1050	1312	4.54				0.009	0.858

Characteristics of two-arm two-stage TB trials where *I* = culture status observed 14 weeks after randomisation and *D* = relapse status at 18 months. Key: for stage *i*, *α*_i_ = significance level, *ω*_i_ = nominal power, *n*_i_ = total sample size required for analysis *i*, *N*_i_ = cumulative number of patients recruited by the end of stage *i*, *t*_i_ = predicted timing (in years) of the end of stage *i*, ESS |*H*_0_ = expected sample size under the null hypothesis, *ρ*|*H*_h_ = correlation between stages under hypothesis *H*_h_, *A*_i_ = probability of passing stage *i* under *H*_0_, *Ω*_i_ = probability of passing stage *i* under *H*_1_.

The efficiency of each design was measured by its expected sample size (ESS), that is, the mean number of patients recruited to the trial before it is terminated [22], calculated under the null hypothesis. ESS was compared between designs with roughly similar overall operating characteristics to determine which is likely to require fewer resources when the experimental treatment is ineffective. For a single-stage trial, such as those in Table 1, the ESS is equal to the overall sample size since there is no opportunity for stopping before the planned end of the trial (except perhaps in extreme circumstances such as overwhelming efficacy of an arm).

To calculate the overall operating characteristics in the seamless designs an estimate of the positive predictive value, that is, the probability of a patient not relapsing or being classed as a treatment failure given that they have a negative culture, was obtained from a meta-analysis by Horne et al. [23] who estimated it to be 95% (95% CI (95%, 96%)) for cultures taken at 2 months. This value was assumed to be the same under *H*_0_ and *H*_1_ for each intermediate outcome.

### Simulation study

Performing an analysis in a MAMS trial which ignores the stopping guidelines for lack-of-benefit may result in biased treatment effect estimates [15]. Choodari-Oskooei et al. [15] investigated the extent of this bias for two-arm multi-stage trials with time-to-event outcomes. For arms stopped at the first interim analysis for lack-of-benefit they showed that on average the estimated treatment effects appeared slightly less effective than their corresponding true values. However, the bias was markedly reduced by continuing to follow-up patients on the intermediate and definitive outcomes and reanalysing the data at the planned end of the trial. For truly effective arms, they showed that the bias in the estimated treatment effects on the definitive outcome at the final stage analysis was of no practical importance.

In the time-to-event case, interim analyses occur when a pre-specified number of events have been observed in the control arm. In arms in which recruitment is stopped early there is scope for continuing to follow-up patients who have not yet experienced the event(s) of interest and including them in a reanalysis at the planned end of the trial to obtain a less biased estimate of the treatment effect. This is also applicable when outcomes are observed at the end of a fixed follow-up period since not all patients will have had both their intermediate and definitive outcomes observed by each interim analysis.

A simulation study was conducted using the two-stage phase II and phase II/III TB trial designs shown in Tables 2 and 3 respectively, to quantify the bias of treatment effects estimated on the definitive outcome at: 

(a) The first interim analysis in arms which are not continued to the second stage

(b) A reanalysis of the same arms (against all control arm patients recruited concurrently) after intermediate and definitive outcome data have been obtained from all patients

(c) The final stage analysis of all arms which pass all intermediate stages

Phase II/III designs in which the follow-up period for *I* was 4 weeks rather than the current 14 weeks (designs not shown) were also used to investigate the effect of follow-up length in (b).

In addition to bias, the proportion of arms for which recruitment is stopped at the first interim analysis and the proportion which continue recruiting to the final stage of the trial, as well as the pairwise type I error rate, power and correlation between stages were determined in the simulations and compared to their corresponding calculated values.

For each design shown in Tables 2 and 3, the bias associated with the following four pairs of underlying treatment effects for the culture status (*CS*) and relapse outcomes (*R*) were investigated in the simulations: 

(A).*θ*_*CS*_=−5*%*, *θ*_*R*_=−10*%* (treatment effects worse than those under *H*_0_)

(B). *θ*_*CS*_=0*%*, *θ*_*R*_=−6*%* (treatment effects under *H*_0_ (see Table 1))

(C). *θ*_*CS*_=8*%*, *θ*_*R*_=−3*%* (treatment effects between those under *H*_0_ and *H*_1_)

(D). *θ*_*CS*_=13*%*, *θ*_*R*_=0*%* (treatment effects under *H*_1_ (see Table 1))

By assessing bias in scenarios (a), (b) and (c) for this variety of treatment effects, recommendations can be made for designing multi-stage trials which reduce bias, thus improving the accuracy of treatment effect estimates which might be used, for example, in future meta-analyses, policy-making decisions or the design of future trials.

#### Simulation methods

To perform the bias assessment and assess the accuracy of the calculation of the pairwise operating characteristics, individual patient data were simulated for each phase II and phase II/III design under treatment effects A-D. In each case 40,000 replicates were generated to estimate pass/fail rates to an accuracy of at least 0.5% at the 5% significance level. For each patient, missing value indicators for the *I* and *D* outcomes were drawn from Bernoulli distributions with parameters derived from Table 1. In the designs where *I*≠*D* the probability of observing the definitive outcome was not conditional on observing the intermediate outcome. Although this reduces the correlation between stages compared to the calculation given in the appendix where all patients with a missing intermediate outcome are also assumed to have a missing definitive outcome, these different assumptions will indicate the robustness of the calculation of the overall type I error rate and power.

Patient outcomes were drawn from Bernoulli distributions with control arm event rates derived from Table 1. The underlying event rates for experimental arms with underlying effects A-D were found by adding on the corresponding treatment effects shown above. Since the phase III outcome (relapse) is dependent on culture status, the event rate will differ according to whether a patient’s culture status is positive (*CS*=0), negative (*CS*=1) or missing. The positive predictive value (PPV= *P*(*R*=1|*CS*=1)) is the relapse event rate for patients with a positive culture status and the estimate from Horne et al. (95%) [23] was assumed for all arms. The probability *P*(*R*=1|*CS*=0) for each treatment arm was then found by rearranging the formula

$$\begin{array}{l} P(R=1)=P(R=1|\text{CS}=1)P(\text{CS}=1)\\ \;+P(R=1|\text{CS}=0)P(\text{CS}=0) \end{array}$$

Unconditional event rates were used for patients with missing intermediate outcomes.

When simulating each trial, analyses were triggered once the pre-determined number of control arm patients had their outcome of interest observed. The pairwise type I error rate and power for each design was calculated as the proportion of arms simulated under *H*_0_ (treatment arm B) and *H*_1_ (treatment arm D) respectively which passed all stages of the trial. For each underlying treatment effect in each design, the absolute bias in scenarios (a), (b) and (c) was calculated as the average deviation of all treatment effect estimates from the true value.

## Results

### Examples of phase II TB trials

Table 2 summarises the sample sizes and durations of phase II two-arm two-stage trials which use culture status at 4 or 8 weeks of follow-up as the primary endpoint for both the intermediate and definitive outcomes. A constant recruitment rate of 200 patients/year was assumed in both stages.

The results show that the maximum sample sizes of the two-stage designs shown in Table 2 are higher than the corresponding fixed sample sizes, however, their expected sample sizes are much lower as they allow recruitment to be stopped early if the experimental treatment does not show sufficient benefit at the first stage. Increasing the power in the first stage reduces the difference between the maximum and fixed sample sizes, however, this also increases the expected sample sizes due to a larger first stage. Thus, a balance needs to be found between the two measures. As expected, the correlation between stages increases as the gap between analyses decreases, however, this only marginally increases the type I error rate and power.

Although designs (ii) and (iv) have similar overall operating characteristics, the design which uses a first stage significance level of *α*_1_=50*%* (design (ii)) has a much smaller expected sample size. On the other hand, designs (i) and (iii) also have similar overall operating characteristics but are approximately equally efficient. Unsurprisingly, the ESS is smaller when using a shorter follow-up period since fewer patients are recruited during the first stage of the trial. All two-stage designs have the same maximum sample size as they use the same final stage operating characteristics.

There appears little advantage in using these two-stage designs over a single-stage design for two-arm phase II TB trials, however, if multiple treatments are to be evaluated in a single trial then stopping guidelines for lack-of-benefit will become much more useful. Due to the current length of follow-up for culture status (8 weeks) and short length of phase II trials, using more than two-stages is unlikely to improve efficiency.

### Examples of seamless phase II/III TB trials

Examples of seamless two-stage TB trials are presented in Table 3. A constant recruitment rate of 200 patients/year was assumed for the intermediate (phase II) stage and a much higher recruitment rate of 800 patients/year was used for the second (phase III) stage. Under these assumptions the maximum duration of each design is no longer than 5 years. If similar recruitment rates are assumed for the fixed sample designs shown in Table 1 then the maximum duration of conducting both trials separately is approximately 8.5 years assuming a modest delay between phases of 3 years. Furthermore, the overall power of the seamless designs (over 80%) is much higher than that for conducting trials separately (68%) and maximum sample sizes are over 100 patients lower.

The between-stage correlations in these designs are much lower than those in the phase II designs for two reasons. Firstly, the positive predictive value is effectively 1 in designs with *I*=*D* (see Appendix) whereas the seamless designs use a slightly lower value (0.95). Secondly, the interim and final analyses are much further apart in terms of sample size than in the phase II designs, which further reduces the correlation. Although not problematic, the immediate consequence of lower between-stage correlation is a reduction in both the pairwise type I error rate and power, and so the stagewise operating characteristics may have to be increased to achieved the desired level for each measure.

A downside of the seamless designs presented in Table 3, as illustrated by the high ESS, is that ineffective arms have a reasonable chance of proceeding to the final stage of the trial due to the high significance level used in the first stage. To combat this, the large gap between the first and final analyses means that an extra intermediate stage could be added to the trial. For example, adding a second intermediate stage with 95% power and a 10% significance level to design (vi) in Table 3 reduces the ESS to 377 with only a 3% reduction in overall power. This loss can be recovered by slightly increasing the stagewise powers. Identifying MAMS designs which maintain the overall operating characteristics but have desirable properties such as minimising the expected or maximum sample sizes is an area of ongoing research.

Clearly there is much more benefit in using the MAMS design for seamless phase II/III TB trials than for phase II alone. We have demonstrated the savings in time and resources that can be achieved in using seamless two-arm two-stage trials over conducting each phase separately. For multi-arm multi-stage seamless trials, the savings will potentially be much greater compared to conducting separate phase II and phase III trials for each experimental treatment.

### Results of simulation study

Table 4 shows that the overall type I error rate, power and correlation between stages estimated from the simulations of the designs shown in Tables 2 and 3 agree very well with the corresponding calculated values. As expected, when *I*≠*D* the correlation between stages estimated from the simulations is slightly lower than the calculated values, however, this leads to only a negligible difference between the overall type I error rates and powers showing that the calculation is robust to the degree of dependence between observing each outcome.

**Table 4:** Correlations, type I error rates and powers obtained from simulation compared to calculated values

**Design**	***α***_**1**_	***ω***_**1**_	**From calculation**				**From simulation**			
			***ρ*** **|** ***H***_**0**_	***ρ*** **|** ***H***_**1**_	***α***	***ω***	$\hat{\rho }|H_{0}$	$\hat{\rho }|H_{1}$	$\hat{\alpha }$	$\hat{\omega }$
* I*=*D*=culture status										
(i)	0.50	0.90	0.39	0.39	0.021	0.826	0.38	0.38	0.021	0.828
(ii)	0.50	0.95	0.50	0.50	0.023	0.870	0.50	0.50	0.024	0.872
(iii)	0.20	0.90	0.65	0.65	0.020	0.843	0.64	0.65	0.019	0.847
(iv)	0.20	0.95	0.76	0.76	0.023	0.883	0.76	0.76	0.023	0.885
* I*= culture status, *D* = relapse										
(v)	0.50	0.90	0.10	0.08	0.015	0.813	0.07	0.06	0.014	0.809
(vi)	0.50	0.95	0.12	0.11	0.015	0.857	0.10	0.09	0.015	0.854
(vii)	0.20	0.90	0.16	0.14	0.008	0.815	0.12	0.11	0.008	0.811
(viii)	0.20	0.95	0.19	0.16	0.009	0.858	0.15	0.12	0.008	0.858

Overall type I error rates, powers and correlations between stages obtained from simulations of designs (i)-(viii) in Tables 2 and 3. Key: *α*_1_ = stage 1 significance level, *ω*_1_ = stage 1 power, *ρ*|*H*_h_ = correlation between stages under hypothesis *H*_h_, *α* = overall type I error rate, *ω* = overall power. Hats indicate values estimated from simulations.

#### Bias in arms dropped at the first analysis

Table 5 summarises the simulation results for the proportion of arms dropped at the end of the first stage and the absolute bias in their treatment effect estimates on the definitive outcome at the interim analysis and after all remaining patients have completed follow-up. The proportion of arms dropped under *H*_0_ (treatment effect B) and *H*_1_ (treatment effect D) is as expected given the significance level and power in this stage.

**Table 5:** Absolute bias in arms dropped at the first interim analysis

			***I* =** ***D* = culture status**				***I* = culture status,** ***D* = relapse**		
***α***_**1**_	**Treatment**	**% stop**	**True** ***θ***_***D***_	**Bias on** ***D***	**Bias on** ***D*** **after f/u**		**True** ***θ***_***D***_	**Bias on** ***D*** **after f/u**	
	**arm**	**at**		**at interim**	**Length of f/u**	**Length of f/u**		**Length of f/u**	**Length of f/u**
		**stage 1**		**analysis**	**on** ***I*** **= 4 wks**	**on** ***I*** = 8 wks*		**on** ***I*** **= 4 wks**	**on** ***I*** = 8 wks*
1 power *ω*_1_ = 90%									
0.5	A	65	-5%	-6.9%	-4.7%	-3.2%	-10%	-1.4%	-1.0%
	B	49	0%	-9.5%	-6.5%	-4.6%	-6%	-1.7%	-1.3%
	C	23	8%	-14.6%	-9.8%	-7.0%	-3%	-2.7%	-1.9%
	D	10	13%	-18.2%	-12.3%	-8.8%	0%	-3.0%	-2.1%
0.2	A	94	-5%	-0.9%	-0.8%	-0.7%	-10%	-0.2%	-0.2%
	B	80	0%	-2.6%	-2.1%	-1.9%	-6%	-0.5%	-0.5%
	C	35	8%	-6.9%	-5.9%	-5.0%	-3%	-1.6%	-1.4%
	D	10	13%	-10.7%	-9.2%	-7.7%	0%	-2.2%	-2.0%
Stage 1 power *ω*_1_ = 95%									
0.5	A	70	-5%	-4.6%	-3.6%	-2.8%	-10%	-1.0%	-0.9%
	B	49	0%	-7.3%	-5.6%	-4.5%	-6%	-1.5%	-1.2%
	C	17	8%	-12.6%	-9.8%	-7.6%	-3%	-2.7%	-2.1%
	D	5	13%	-16.3%	-12.9%	-9.9%	0%	-3.4%	-2.7%
0.2	A	95	-5%	-0.6%	-0.6%	-0.5%	-10%	-0.2%	-0.2%
	B	80	0%	-2.1%	-1.8%	-1.6%	-6%	-0.5%	-0.4%
	C	27	8%	-6.7%	-6.0%	-5.3%	-3%	-1.7%	-1.5%
	D	5	13%	-10.8%	-9.6%	-8.4%	0%	-2.3%	-2.1%

Simulation results showing the proportion of trials stopped at the first interim analysis and the absolute bias for such arms in the estimated treatment effect on *D* at the interim analysis and after all remaining patients have been followed up. Key: *α*_1_ = significance level in stage 1, *θ*_D_ = underlying treatment effect on the definitive outcome. *Plus an additional 6 week delay to determine culture status.

The results show that, on average, treatment effects are underestimated in arms which do not show sufficient benefit at the first interim analysis. When *I*=*D* the absolute bias in such arms is particularly high when a high significance level (50%) and relatively low power (90%) is used (design (i) in Table 2), in other words, the earlier the interim analysis occurs. In this design the magnitude of the absolute bias is over 9% under *H*_0_. However, the bias is markedly reduced in a reanalysis after all remaining patients have had their outcome observed, with a greater reduction in bias when using a longer follow-up period or, more generally, when more patients can be added to the reanalysis. In this particular example, the magnitude of the absolute bias under *H*_0_ decreases from 9.5% to 6.5% for 4 week follow-up and to 4.6% if outcome observation is delayed by 14 weeks after randomisation.

When using a relatively low significance level in the first stage (e.g. 20%) the bias is of no practical importance in arms which are likely to be stopped at that analysis, particularly after follow-up is complete. When *I*≠*D*, the bias in the treatment effect estimates for *D* is much lower than when the same outcome is used throughout the trial, even when using a high significance level in the first stage.

#### Bias in arms reaching the final analysis

Table 6 shows that treatment effects estimated at the final planned analysis of the trial are overestimated on average, although the bias is generally not as large as it is for arms dropped at the first analysis. The results suggest that bias decreases the further the interim analysis is in terms of sample size from the final analysis (i.e. as the correlation between stages decreases) and when the chance of proceeding to the final stage of the trial is higher, as is the case for effective arms.

**Table 6:** Absolute bias in arms reaching the final analysis

***α***_**1**_	**Treatment**	***θ***_***D***_	***ω***_**1**_**=0** ***.*** **90**			***ω***_**1**_**=0** ***.*** **95**		
	**arm**		**% Pass**	$E({\hat{\theta }}_{D})$	***b***_***D***_	**% Pass**	$E({\hat{\theta }}_{D})$	***b***_***D***_
* I* = *D* = culture status at 8 weeks								
0.5	A	-5%	35	-3.1%	1.9%	29	-2.2%	2.8%
	B	0%	51	1.4%	1.4%	50	1.8%	1.8%
	C	8%	78	8.6%	0.6%	83	8.7%	0.7%
	D	13%	90	13.3%	0.3%	95	13.2%	0.2%
0.2	A	-5%	6	0.9%	5.9%	5	2.4%	7.4%
	B	0%	20	4.2%	4.2%	20	4.8%	4.8%
	C	8%	65	9.5%	1.5%	73	9.5%	1.5%
	D	13%	90	13.5%	0.5%	95	13.4%	0.4%
* I* = culture status at 8 weeks, *D* = relapse								
0.5	A	-10%	35	-9.8%	0.2%	30	-9.7%	0.3%
	B	-6%	51	-5.9%	0.1%	51	-5.8%	0.2%
	C	-3%	77	-3.0%	0.0%	83	-2.9%	0.1%
	D	0%	90	0.0%	0.0%	95	0.0%	0.0%
0.2	A	-10%	6	-9.4%	0.6%	5	-9.3%	0.7%
	B	-6%	20	-5.6%	0.4%	20	-5.5%	0.5%
	C	-3%	65	-2.9%	0.1%	73	-2.9%	0.1%
	D	0%	90	0.0%	0.0%	95	0.0%	0.0%

Simulation results showing the proportion of trials which continue to the final stage of the trial (% pass) and the absolute bias in the estimated treatment effect on *D* at the final analysis. Key: *θ*_*D*_ = underlying treatment effect on the definitive outcome, *α*_1_ = significance level in stage 1, *ω*_1_ = nominal power in stage 1,$\text{E}({\hat{\theta }}_{\text{D}})$ = average treatment effect on the definitive outcome in the final stage,${\text{b}}_{\text{D}}=\text{E}({\hat{\theta }}_{\text{D}})-\theta_{\text{D}}$ = bias in the average treatment effect estimate on the definitive outcome in the final stage.

In the examples used in Table 6 the bias is practically zero in all cases when *I*≠*D*, even for ineffective arms. This is due to the very low correlation between stages in these designs (roughly 0.1). However, even when the correlation is higher, for example when *I*=*D*, the bias is still approximately zero for arms which are likely to proceed to the final stage. Bias is higher for ineffective arms, however, in a well-designed MAMS trial such arms should have little chance of reaching the final stage.

## Discussion

We have successfully adapted the MAMS design initially developed by Royston et al. [6] to binary outcomes which are observed at the end of a fixed follow-up period and analysed using an absolute difference in proportions. Throughout this paper we have used TB as an example of a disease area where a MAMS approach could dramatically speed up treatment evaluation compared to the traditional approach of separate, two-arm phase II and III trials. Savings in time and resources are particularly large when using the MAMS design to incorporate both phase II and phase III into a single seamless trial, however, savings are still likely to be made when using it to design multi-arm phase II trials. Many new and repurposed drugs are currently in clinical development for TB and so a huge number of new regimens are likely to be available for testing in phase II and III trials in the near future. Evaluating them in separate, single stage trials will not only be costly but will prolong the discovery of a simpler and shorter effective regimen by decades. Use of novel trial designs such as the MAMS design is therefore urgently required.

Further work is needed to determine the best intermediate outcome for long-term relapse before the MAMS design described here can be used to evaluate TB treatments in a seamless phase II/III trial. The methods used by Barthel et al. [14], who evaluated the performance of the MAMS design for time-to-event outcomes in four cancer trials, could be applied to past TB trials. If the rate at which trials are incorrectly stopped for lack-of-benefit on culture status at eight weeks is high then other intermediate outcomes will need considering, such as culture status at other time points. Another candidate for the intermediate outcome is time to culture conversion, which is increasingly being used in phase II trials and is arguably a more reliable surrogate endpoint than culture status at a single time point [24]. Although surrogacy is not a requirement for an intermediate outcome it is likely that a surrogate outcome will be a reliable choice. An ongoing trial conducted by the PanACEA consortium with a MAMS design (ClinicalTrials.gov identifier NCT01785186) is using this endpoint but since this is a phase II trial the definitive outcome is also time to culture conversion. Incorporating this outcome into a MAMS design with a binary definitive outcome will require further extensions to the methodology which we are currently developing.

The amount of bias likely to be generated in various examples of phase II and phase II/III TB trials was investigated and was shown to be of no practical importance in arms reaching the final analysis, particularly in effective arms or when treatment selection is based on an intermediate outcome. In general, the bias at the final analysis increases as the treatment effects estimated at each stage become more correlated. This is caused by having short stage durations in which only a small amount of new data can be collected. Ensuring that stages are adequately spaced is not only practical from the perspective of everyone involved in the trial but it will also limit the amount of bias likely to be generated.

As shown by Choodari-Oskooei et al. [15], we also found that having an early first interim analysis increased the bias of treatment effect estimates in arms dropped at this analysis, particularly when the intermediate and definitive outcomes were identical. Bias was markedly reduced in a reanalysis after all patients had completed follow-up. It should be noted, however, that the average treatment effect in arms which are stopped early for lack-of-benefit (i.e. are statistically non-significant) will necessarily appear less effective than their true value [25]. Freidlin and Korn [26] suggest that the most appropriate comparator for the *x%* of trials stopped at the first interim analysis is the average treatment effect estimate of the same outcome in the corresponding *x%* most extreme trials in the fixed sample-size design (the design that has no interim analyses). When taking this into consideration the bias estimates in Table 5 are nearly halved (data not shown).

A calculation for the overall type I error rate for a single experimental arm was described, thus allowing control of this measure. However, in a multi-arm trial it may be more important to control the familywise type I error rate (FWER), that is, the probability of rejecting at least one true null hypothesis at the end of the trial. Freidlin et al. [7] argue that this decision depends on the clinical questions that the trial is addressing. For example, if a multi-armed trial was used purely for efficiency reasons and the interpretation of the results of one arm has no influence over the results of other arms then they argue that no control of the FWER needs to be made. On the other hand, if in some way the treatment arms are related, such as different doses or schedules of the same treatment, then multiplicity adjustment should be made. Others have said that if a multi-arm design is to be used in a confirmatory trial then FWER control is a requirement [27,28].

For the MAMS design described here, a crude method for ensuring that the FWER is no higher than some prespecified level is to apply a Bonferonni correction to the pairwise type I error rate: i.e. in a trial with *K* experimental arms, a pairwise *α* equal to FWER/*K* could be used. However, such a correction can be too conservative and may result in a trial which is much larger than might be necessary, thus losing efficiency. More accurate methods for controlling the FWER in the strong sense (i.e. under any parameter configuration) are therefore required and is a subject of ongoing research. Alternatively, other MAMS designs which allow stopping for lack-of-benefit and control the FWER are available [29-32].

## Conclusions

The methodology presented in this paper is aimed at reducing the amount of time and resources required to obtain reliable results from clinical studies. A Stata program for designing MAMS trials with binary outcomes is available from the authors upon request. Further work is ongoing into finding MAMS designs which are the most efficient in terms of the expected or maximum sample size or a mixture of the two for a given overall pairwise or familywise type I error rate and power. In TB, the MAMS design will have the greatest impact in phase II/III seamless designs, however, considerable savings are also likely to be made in other disease areas.

## Appendix

### Appendix: Estimating the correlation matrices

Before *A*_*i*_ and *Ω*_*i*_ can be calculated the correlation matrices,$R_{i}^{0}$ and$R_{i}^{1}$, whose (*j*,*k*)th entries are the correlations between the treatment effects in stages *j* and *k* under *H*_0_ and *H*_1_ respectively, are required. We begin with a general case where the binary outcomes of interest in stages *j* and *k* are different. Suppose outcome *X* is the outcome of interest in stage *j* and outcome *Y* is of interest in stage *k* with *j*<*k* and denote the observed treatment effects by${\hat{\theta }}_{j}={\hat{\pi }}_{j}^{E}-{\hat{\pi }}_{j}^{C}$ and${\hat{\theta }}_{k}={\hat{\pi }}_{k}^{E}-{\hat{\pi }}_{k}^{C}$ respectively.

If$\pi_{i}^{h}=\pi_{i}^{C}+\theta_{i}^{h}$ is the target experimental arm event rate under hypothesis *H*_*h*_ then the standard deviation of$\theta_{i}^{h}$ in its normal approximation is

$$\sigma_{i}^{h}=\sqrt{\frac{\pi_{i}^{h}\left(1-\pi_{i}^{h}\right)}{{\text{An}}_{i}^{C}}+\frac{\pi_{i}^{C}\left(1-\pi_{i}^{C}\right)}{n_{i}^{C}}}$$

Assuming success rates between treatment arms are independent, the correlation between${\hat{\theta }}_{j}$ and${\hat{\theta }}_{k}$ under hypothesis *H*_*h*_ (*h*=0,1), denoted by$\rho_{\left(j,k\right)}^{h}$, is

$$\begin{array}{lll} \rho_{\left(j,k\right)}^{h} & =\frac{\text{Cov}({\hat{\theta }}_{j},{\hat{\theta }}_{k})}{\sigma_{j}^{h}\sigma_{k}^{h}}\; & \;\\ & =\frac{\text{Cov}\left({\hat{\pi }}_{j}^{h}-{\hat{\pi }}_{j}^{C},{\hat{\pi }}_{k}^{h}-{\hat{\pi }}_{k}^{C}\right)}{\sigma_{j}^{h}\sigma_{k}^{h}}\; & \;\\ & =\frac{\text{Cov}\left({\hat{\pi }}_{j}^{h},{\hat{\pi }}_{k}^{h}\right)+\text{Cov}\left({\hat{\pi }}_{j}^{C},{\hat{\pi }}_{k}^{C}\right)}{\sigma_{j}^{h}\sigma_{k}^{h}}\; & \; \end{array}$$

Denote by$X_{m}^{C}$ and$Y_{m}^{C}$ the observed *X* and *Y* outcomes respectively for the *m*th patient in the control arm ($X_{m}^{C},Y_{m}^{C}\in \{0,1\}$) where$X_{m}^{C}$ is observed during or before stage *j* and$Y_{m}^{C}$ is observed during or before stage *k* (*j*<*k*). The covariance between the control arm event rates in stage *j* on the *X* outcome and stage *k* on the *Y* outcome is

$$\begin{array}{lll} \;\text{Cov}({\hat{\pi }}_{j}^{C},{\hat{\pi }}_{k}^{C}) & =\text{Cov}\left(\frac{1}{n_{j}^{C}}\overset{n_{j}^{C}}{\underset{l=1}{\sum }}X_{l}^{C},\frac{1}{n_{k}^{C}}\overset{n_{k}^{C}}{\underset{m=1}{\sum }}Y_{m}^{C}\right)\; & \;\\ & =\frac{1}{n_{j}^{C}n_{k}^{C}}\overset{n_{j}^{C}}{\underset{l=1}{\sum }}\overset{n_{k}^{C}}{\underset{m=1}{\sum }}\text{Cov}\left(X_{l}^{C},Y_{m}^{C}\right)\; & \;\\ & =\frac{1}{n_{j}^{C}n_{k}^{C}}\;\overset{n_{j}^{C}}{\underset{l=1}{\sum }}\overset{n_{k}^{C}}{\underset{m=1}{\sum }}\;\left\{E(X_{l}^{C}Y_{m}^{C})-E(X_{l}^{C})E(Y_{m}^{C})\right\}\; & \; \end{array}$$

Assuming observations from different patients are independent implies$E(X_{l}^{C}Y_{m}^{C})=E(X_{l}^{C})E(Y_{m}^{C})$ if *l*≠*m* and so

$$\begin{array}{lll} \text{Cov}({\hat{\pi }}_{j}^{C},{\hat{\pi }}_{k}^{C}) & =\frac{1}{n_{j}^{C}n_{k}^{C}}\overset{n_{j}^{C}}{\underset{l=1}{\sum }}\left(E(X_{l}^{C}Y_{l}^{C})-E(X_{l}^{C})E(Y_{l}^{C})\right)\; & \;\\ & \;\text{since}\;j<k\; & \;\\ & =\frac{1}{n_{j}^{C}n_{k}^{C}}\overset{n_{j}^{C}}{\underset{l=1}{\sum }}\left(\pi_{\left(j,k\right)}^{C}-\pi_{j}^{C}\pi_{k}^{C}\right)\; & \;\\ & =\frac{1}{n_{k}^{C}}\left(\pi_{\left(j,k\right)}^{C}-\pi_{j}^{C}\pi_{k}^{C}\right)\; & \; \end{array}$$

where$\pi_{\left(j,k\right)}^{C}$ is the probability of a patient experiencing both the *X* and *Y* outcomes in the control arm. A similar argument for the covariance of event rates between stages in an experimental arm under *H*_*h*_ gives

$$\text{Cov}\left({\hat{\pi }}_{j}^{E},{\hat{\pi }}_{k}^{E}\right)=\frac{1}{{\text{An}}_{k}^{C}}\left(\pi_{\left(j,k\right)}^{h}-\pi_{j}^{h}\pi_{k}^{h}\right).$$

It follows that

(3) $$\rho_{\left(j,k\right)}^{h}=\frac{\left(\pi_{\left(j,k\right)}^{h}-\pi_{j}^{h}\pi_{k}^{h}\right)+A\left(\pi_{\left(j,k\right)}^{C}-\pi_{j}^{C}\pi_{k}^{C}\right)}{{\text{An}}_{k}^{C}\sigma_{j}^{h}\sigma_{k}^{h}}$$

The values$\pi_{\left(j,k\right)}^{C}$ and$\pi_{\left(j,k\right)}^{h}$ may be estimated from prior knowledge or, if estimates of the positive predictive value in each arm are available, that is, the probability of a patient having a *Y* event given that they have had an *X* event, then from the definition of conditional probability

$$\pi_{\left(j,k\right)}^{C}=P\left(Y_{m}^{C}=1|X_{m}^{C}=1\right)\pi_{j}^{C}$$

and

$$\pi_{\left(j,k\right)}^{h}=P\left(Y_{m}^{h}=1|X_{m}^{h}=1\right)\pi_{j}^{C}.$$

If the outcomes of interest in stages *j* and *k* are the same then equation (3) simplifies. Clearly the positive predictive value is now 1 and so$\pi_{\left(j,k\right)}^{C}=\pi_{j}^{C}$ and$\pi_{\left(j,k\right)}^{h}=\pi_{j}^{h}$. Then

(4) $$\begin{array}{lll} \rho_{\left(j,k\right)}^{h} & =\frac{\left(\pi_{j}^{h}-{\left(\pi_{j}^{h}\right)}^{2}\right)+A\left(\pi_{j}^{C}-{\left(\pi_{j}^{C}\right)}^{2}\right)}{{\text{An}}_{k}^{C}\sigma_{j}^{h}\sigma_{k}^{h}}\; & \;\\ & =\frac{\pi_{j}^{h}\left(1-\pi_{j}^{h}\right)+A\pi_{j}^{C}\left(1-\pi_{j}^{C}\right)}{{\text{An}}_{k}^{C}\sigma_{j}^{h}\sigma_{k}^{h}}\; & \;\\ & =\frac{n_{j}^{C}{\left(\sigma_{j}^{h}\right)}^{2}}{n_{k}^{C}\sigma_{j}^{h}\sigma_{k}^{h}}=\sqrt{\frac{n_{j}^{C}}{n_{k}^{C}}}\; \end{array}$$

since underlying treatment effects are assumed to be constant throughout the trial. Note that these correlations are the same under *H*_0_ and$H_{1}\;(\rho_{\left(j,k\right)}^{0}=\rho_{\left(j,k\right)}^{1})$.

The entries,$\rho_{\left(j,k\right)}^{h}$, below the main diagonal of the correlation matrices can now be calculated using (3) for the correlations between the intermediate and final outcomes and (4) for the correlations between the intermediate outcome in different stages. Since each matrix is symmetric we set$\rho_{\left(j,k\right)}^{h}=\rho_{\left(k,j\right)}^{h}$ and all diagonal entries, i.e. the correlation between treatment effects in the same stage, are$\rho_{\left(j,j\right)}^{h}=1$.

## Abbreviations

MAMS: Multi-arm multi-stage; TB: Tuberculosis; ESS: Expected sample size.

## Competing interests

The authors declare that they have no competing interests.

## Authors’ contributions

DJB drafted the manuscript, carried out the mathematical calculations and performed the simulations. All authors read and approved the final manuscript.

## Pre-publication history

The pre-publication history for this paper can be accessed here:

http://www.biomedcentral.com/1471-2288/13/139/prepub
